# In Situ Transformation of Electrospun Nanofibers into Nanofiber-Reinforced Hydrogels

**DOI:** 10.3390/nano12142437

**Published:** 2022-07-16

**Authors:** Alma Martin, Jenny Natalie Nyman, Rikke Reinholdt, Jun Cai, Anna-Lena Schaedel, Mariena J. A. van der Plas, Martin Malmsten, Thomas Rades, Andrea Heinz

**Affiliations:** 1 LEO Foundation Center for Cutaneous Drug Delivery, Department of Pharmacy, University of Copenhagen, 2100 Copenhagen, Denmark; alma.martin@nu.edu.kz (A.M.); jenny.n.nyman@gmail.com (J.N.N.); rixxd95@live.dk (R.R.); jun.cai@sund.ku.dk (J.C.); anna-lena.schaedel@sund.ku.dk (A.-L.S.); mariena.van_der_plas@sund.ku.dk (M.J.A.v.d.P.); martin.malmsten@sund.ku.dk (M.M.); thomas.rades@sund.ku.dk (T.R.); 2 School of Medicine, Nazarbayev University, Nur-Sultan 010000, Kazakhstan; 3 Division of Dermatology and Venereology, Department of Clinical Sciences Lund, Lund University, S-22184 Lund, Sweden; 4 Department of Physical Chemistry, Lund University, 22100 Lund, Sweden

**Keywords:** biomaterial, coaxial electrospinning, composite material, mechanical properties, tissue engineering, wound healing

## Abstract

Nanofiber-reinforced hydrogels have recently gained attention in biomedical engineering. Such three-dimensional scaffolds show the mechanical strength and toughness of fibers while benefiting from the cooling and absorbing properties of hydrogels as well as a large pore size, potentially aiding cell migration. While many of such systems are prepared by complicated processes where fibers are produced separately to later be embedded in a hydrogel, we here provide proof of concept for a one-step solution. In more detail, we produced core-shell nanofibers from the natural proteins zein and gelatin by coaxial electrospinning. Upon hydration, the nanofibers were capable of directly transforming into a nanofiber-reinforced hydrogel, where the nanofibrous structure was retained by the zein core, while the gelatin-based shell turned into a hydrogel matrix. Our nanofiber-hydrogel composite showed swelling to ~800% of its original volume and water uptake of up to ~2500% in weight. The physical integrity of the nanofiber-reinforced hydrogel was found to be significantly improved in comparison to a hydrogel system without nanofibers. Additionally, tetracycline hydrochloride was incorporated into the fibers as an antimicrobial agent, and antimicrobial activity against *Staphylococcus aureus* and *Escherichia coli* was confirmed.

## 1. Introduction

Designing novel biomaterials with multiple functionalities for skin regeneration purposes is challenging. Among currently available types of biomaterials, hydrogels [1,2] and nanofiber scaffolds [2,3,4] are the most investigated and promising approaches. Hydrogels are three-dimensional structures obtained by swelling of a polymer in water, in some cases after physical or chemical crosslinking [5]. Such systems exhibit a high water content and large porous structure, with pore sizes typically between 20 and 500 µm, depending on the polymer type, polymer concentration and preparation conditions [6] and allowing for migration of fibroblasts [7,8]. However, hydrogels often only show poor mechanical strength and flexibility, which not only makes their handling difficult but also makes them prone to wear and tear [9]. In recent years, self-gelling hydrogels for tissue engineering purposes have been developed, for instance, for bone regeneration. Such systems rely for instance on the ionic gelation of gellan gum solution after addition of bioactive glass particles, where crosslinking of gellan gum is initiated by the release of Ca^2+^ ions, leading to hydrogel formation [10]. Research has also focused on double- and multi-network hydrogels to overcome challenges related to the low mechanical stability of hydrogels. Multi network hydrogels can, for instance, be produced by 3D printing and offer advantageous properties such as self-healing, self-assembly and shear thinning [11]. Double-network gel wound dressings are characterized by a heterogeneous structure in which a firm, strongly cross-linked layer is combined with a slightly cross-linked layer that is stretchable and ensures the integrity of the dressing even under mechanical stress [12]. A different approach to improve the mechanical properties is based on the preparation of nanofiber-reinforced hydrogels [13,14]. Nanofiber scaffolds are often fabricated by electrospinning, which relies on applying a high voltage to a polymer solution to produce dry and flat fiber scaffolds on an oppositely charged collector [15]. Such scaffolds exhibit high porosities but small pore sizes in the low micrometer range, generally between 5 µm and 50 µm, depending on the polymer type, concentration and preparation conditions [16,17]. Nanofibers have been shown to provide mechanical support for cells, facilitating cell migration across the wound bed [3,4,18,19]. Therefore, a combination of these two systems is promising with respect to achieving the desired properties within a single biomaterial [13,14].

The most commonly used strategies to obtain nanofiber-hydrogel composites include multi-step processes involving the separate fabrication of nanofiber scaffolds and hydrogels followed by their combination to form a composite material [13,14]. In this context, nanofiber scaffolds can be used either as untreated or freeze-dried mats and can also be broken into shorter pieces by homogenization or sonication and added to a pre-cursor hydrogel solution, which is subsequently cured and transformed into a hydrogel [7,20,21,22,23,24,25,26]. Another method involves electrospinning nanofibers directly into the precursor hydrogel solution to achieve a similar effect [27,28,29]. However, the direct transformation of electrospun nanofibers into nanofiber-hydrogel composites, i.e., nanofiber-reinforced hydrogels, has not been described yet. Such an approach would result in a material which converts into a nanofiber-reinforced hydrogel in situ, hence not requiring any incorporation of the fibers into a hydrogel prior to its application. Another benefit of this approach would be that a potential wound dressing precisely fills the shape of the wound upon in situ transformation into a hydrogel, which may be beneficial for the treatment of deep wounds [30,31].

Therefore, the aim of this study was to produce core-shell nanofibrous scaffolds that transform into a nanofiber-reinforced hydrogel upon contact with water. To achieve this, coaxial electrospinning was employed, where a core and a shell solution are spun simultaneously to form core-shell nanofibers [32]. Aqueous ethanol and diluted acetic acid (AA), i.e., solvents that are generally recognized as safe [33], were used for electrospinning. The plant protein zein was selected to form the core of the fibers together with small amounts of polyethylene oxide (PEO) to facilitate electrospinning [34], whereas the animal-derived protein gelatin was used as a shell polymer. Both zein and gelatin are biocompatible and biodegradable [35,36]. Hydrophobic zein is insoluble in water and is promising for electrospinning drug delivery systems for tissue engineering [35]. Gelatin is a degradation product of collagen and carries arginine–glycine–aspartate (RGD) motifs that are beneficial for cell attachment and proliferation [37]. Due to its hydrophilicity, crosslinking agents such as glutaraldehyde or polydopamine (PDA) are needed to prevent instant disintegration of electrospun gelatin-based materials in water [36,38,39,40]. PDA, a versatile non-toxic polymer obtained through oxidation of dopamine hydrochloride upon exposure to UV light or oxidants such as vapors of ammonium carbonate [41,42], was used in this study. It has recently gained significance as an adhesive antimicrobial coating material that binds to various inorganic and organic materials and can be formulated into drug delivery systems [43]. Furthermore, polyethylene imine (PEI), a cationic polymer with the ability to disrupt bacterial cell walls and membranes through pore formation, was crosslinked into the gelatin-PDA matrix in this study to confer antimicrobial activity to the fibers [41,44,45,46]. Due to its excellent gene encapsulation efficiency and its intrinsic endosomolytic activity, PEI is widely used for gene delivery purposes [47]. PEI exists in a linear or branched form and shows a concentration-dependent cytotoxicity for fibroblasts, keratinocytes and other cell types through membrane disruption, which, however, can be decreased by crosslinking it onto electrospun gelatin matrices while retaining its antimicrobial activity [41]. In addition, the broad-spectrum antibiotic tetracycline hydrochloride was incorporated into one of the fiber scaffolds as a model drug for comparison. While fiber scaffolds from a combination of gelatin, PEI and PDA [41], as well as a combination of gelatin and PDA [38,40], have been previously reported, toxic organic solvents, such as 2,2,2-trifluoroethanol, and a uniaxial set-up were used for electrospinning in these studies, i.e., these fibers did not contain a core polymer. With respect to coaxial electrospinning, a single study has been carried out, which deals with gelatin-zein core-shell fibers containing gelatin in the core for encapsulation of polyphenolic antioxidants for food science purposes [48].

Compared to these previous investigations, the aim of this study was to obtain a novel perspective on core-shell zein-based fibers capable of in situ transformation to fiber-reinforced hydrogels. Overall, we successfully demonstrate in this study that zein-gelatin core-shell nanofibers can be produced by coaxial electrospinning and can be transformed into hydrogels in situ upon contact with water. The nanofiber-reinforced hydrogels display improved mechanical properties compared to uniaxially electrospun nanofiber scaffolds from pristine gelatin, which turn into hydrogels that are not reinforced by zein fibers. Potent antimicrobial activity of the nanofiber-reinforced hydrogels is related to both the immediate release of tetracycline hydrochloride and the antimicrobial activity of PEI.

## 2. Materials and Methods

### 2.1. Materials

All materials were purchased from Sigma Aldrich (Sigma-Aldrich Inc., Darmstadt, Germany), unless specified differently. Zein (~19 kDa–22 kDa), polyethylene oxide (PEO, 900 kDa), gelatin from porcine skin (type A), dopamine hydrochloride, branched PEI (~25 kDa), ammonium carbonate, tetracycline hydrochloride (T, ~95% purity), antimicrobial susceptibility test discs (30 µg T, Oxoid, Roskilde, Denmark), absolute ethanol (VWR International, as part of Avator, Søborg, Denmark) and glacial acetic acid (≥99%) (AA) were used. Milli-Q water was obtained from a Reference A+ water purification dispenser (Merck, Darmstadt, Germany). Luria-Bertani (LB) broth and Bacto Agar were purchased from Saveen & Werner AB (Limhamn, Sweden).

### 2.2. Electrospinning of Nanofiber Scaffolds

**Figure 1:**
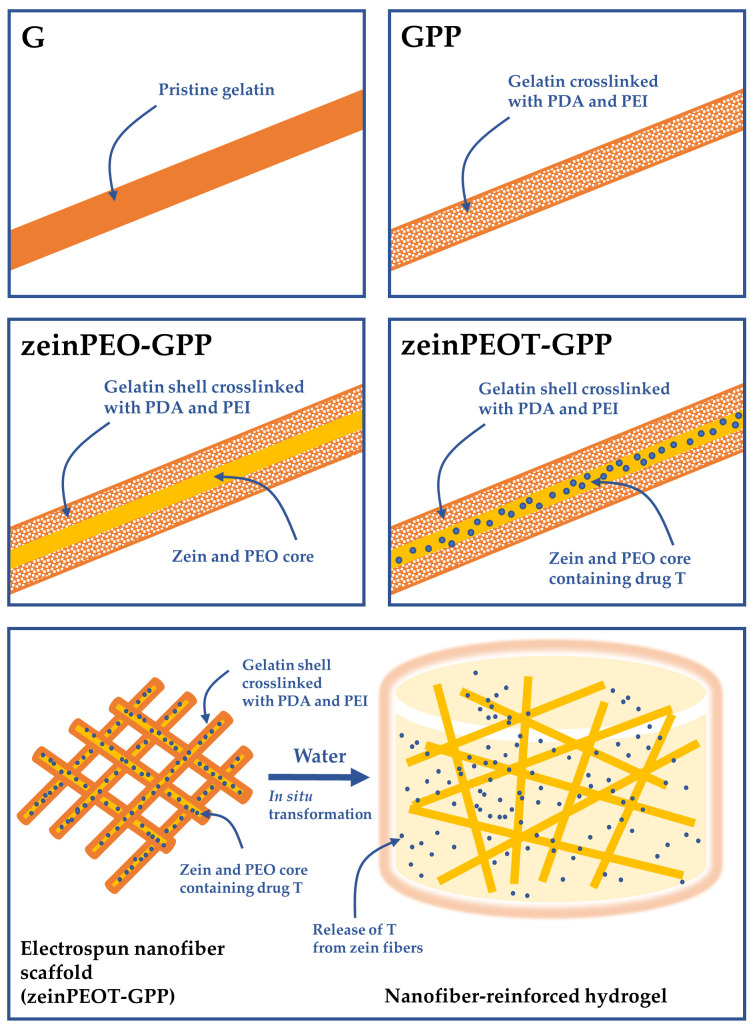
Different types of uniaxial and coaxial samples that were electrospun in the scope of this study (top four images) and concept of the in situ transformation of the nanofiber scaffold into a nanofiber-reinforced hydrogel (bottom image). Key: G, sample containing gelatin; GPP, sample containing gelatin crosslinked by polydopamine and polyethyleneimine; zeinPEO-GPP, sample containing zein and polyethylene oxide in the core and gelatin crosslinked by polydopamine and polyethyleneimine in the shell; zeinPEOT-GPP, sample containing zein, polyethylene oxide and tetracycline hydrochloride in the core and gelatin crosslinked by polydopamine and polyethyleneimine in the shell.

Different gelatin-containing samples were produced by uniaxial and coaxial electrospinning at 25 °C and 45% relative humidity. The electrospinning conditions were selected according to the stability of the electrospinning process and are summarized in Table 1. The uniaxial samples were a PDA-PEI-crosslinked gelatin sample (GPP) and an un-crosslinked gelatin sample (G) as a reference. The coaxial samples both contained zein in the core and PDA-PEI-crosslinked gelatin in the shell, either without (zeinPEO-GPP) or with T in the core (zeinPEOT-GPP) (Figure 1).

For uniaxial electrospinning of GPP, 40% (*w*/*v*) gelatin was dissolved in 60% (*v*/*v*) aqueous AA in a sonication bath at 80 °C for 1 h and then continuously stirred for 1 h at 80 °C. After the solution cooled down to RT, 5% (*w*/*w* of gelatin) PEI and 2% (*w*/*w* of gelatin) dopamine HCl were added and allowed to mix overnight under shaking at 150 rpm at 25 °C. The control sample G contained pristine gelatin without PEI, dopamine HCl and T. For coaxial electrospinning (samples zeinPEO-GPP and zeinPEOT-GPP), 20% (*w*/*v*) of zein and 5% (*w*/*w* of zein) PEO were used as a core solution using 80% (*v*/*v*) aqueous ethanol as a solvent. Zein was added 2 h prior to electrospinning and dissolved at 60 °C. The composition of GPP from the uniaxial set-up was used as a shell solution. For drug-loaded fibers, 5% (*w*/*w* of zein) T was added to the core solution and mixed for 10 min. 

After electrospinning, all samples except for G were crosslinked with ammonium carbonate vapors in a sealed desiccator with 5 g of ammonium carbonate for 48 h. All prepared fiber mats were stored at 0% relative humidity and RT and were sterilized with UV light at 254 nm for 2 h on each side prior to further analysis.

### 2.3. Morphological Characterization of Nanofiber Scaffolds

Sample punches of 6 mm in diameter were coated with gold (Sputter coater Cressington 108 auto, Ted Pella Inc., Redding, CA, USA) for 15 s and analyzed by scanning electron microscopy (SEM) on a TM3030 (Hitachi, Tokyo, Japan) for fiber diameters and fiber distribution. Tests were conducted in triplicate. All images were analyzed with ImageJ software, DiameterJ plugin (1.52a version, National Institutes of Health, Bethesda, MD, USA) [49]. At least 100 fibers of each sample type were examined for fiber diameter and size distribution.

### 2.4. Interactions of Nanofiber Scaffolds with Water

Wettability (water contact angle), water uptake and mass loss were determined for all samples (6 mm diameter punches). The wettability of the samples was analyzed using a Drop Shape Analyzer (DSA100, Krüss, Hamburg, Germany) at RT for 50 s using the sessile drop method. For water uptake and mass loss experiments, the samples were incubated in a 24-well plate in Milli-Q water at 37 °C under shaking at 200 rpm (neoMix thermoshaker, neoLab, Heidelberg, Germany) for 24 h. The samples were removed from the wells at each time point, gently blotted with tissue paper and sample weight and dimensions were recorded with a digital caliper to determine the water uptake and volume change, respectively. For mass loss experiments, samples were dried for 24 h and their dry weight was measured. Additionally, the swollen samples were quenched with liquid nitrogen, freeze-dried (Christ Epsilon 2-4 LSC, Osterode, Germany) for 24 h and analyzed with SEM for morphology and pore size using Image J. Water vapor sorption and desorption profiles were determined using a vapor sorption analyzer (VTI-SA+, TA instruments, New Castle, NY, USA). Samples were dried at 60 °C at a heating rate of 2 °C min^−1^ at 0% relative humidity and then subjected to gradual increase in relative humidity up to 90% at a constant temperature of 25 °C. All analyses were conducted in triplicate.

### 2.5. Mechanical Characterization of Nanofiber Scaffolds

Mechanical properties of the nanofiber scaffolds were examined on a texture analyzer TA.XT plus (Stable Micro Systems, Godalming, UK) at a force of 0.01 N and a speed of 0.5 mm s^−1^ in quintuplicate as described previously [34]. In addition, texture profile analysis was carried out to determine the compression behavior of the samples, which were cut into pieces of 10 mm in diameter. Texture profile analysis was carried out at 0.05 N force, 50% strain, with 0.5 mm s^−1^ test and post-test speeds and 30 s of contact time. Hardness was determined as the force required for a 40% deformation of the sample, while springiness and cohesiveness refer to the sample’s ability to spring back to its original shape as measured by the texture analyzer after the first and second compression, respectively [27]. 

### 2.6. Solid-State Characterization of Nanofiber Scaffolds

Solid-state characterization of the electrospun fiber mats and their starting materials was performed using thermogravimetric analysis (TGA) and X-ray diffraction (XRD). TGA was performed using a TGA 5500 (New Castle, DE, USA). For TGA, all samples were placed in pre-tared platinum pans and heated with a rate of 10 °C min^−1^ from 50 °C to 395 °C. XRD patterns were obtained using an X’pert PRO (PANanalytical, Malvern, UK). All samples were scanned in the range of 5–35° (2θ) at 45 kV and 40 mA. Reference diffractograms for tetracycline hydrochloride were obtained from CCDC database (ACHRMY), and reference diffractograms for PDA [50] and PEO [51] were taken from the literature. TGA and XRD measurements were conducted in triplicate.

### 2.7. Drug Loading and Release

For drug loading experiments, sample punches (6 mm diameter) were dissolved in 2 mL of AA. After filtering the samples through 0.22 µm syringe filters, the samples were measured using UV-Vis spectrophotometry (Shimadzu UV-1900, Kyoto, Japan) at 354 nm. Drug-free samples were used as a control, and all experiments were carried out in triplicate. Drug release experiments were carried out on sample punches (6 mm diameter), which were placed in a 24-well plate in 2 mL water at 37 °C. The samples were shaken at 200 rpm (neoMix thermoshaker, neoLab, Heidelberg, Germany) for the duration of the experiments. At different time points (0 min, 5 min, 10 min, 20 min, 30 min, 1 h, 3 h, 24 h and 48 h), 1 mL was removed from the well plates and replaced with 1 mL fresh 37 °C Milli-Q water. The removed samples were filtered through a 0.22 μm syringe filter and analyzed by UV-Vis spectrophotometry (Shimadzu UV-1900, Kyoto, Japan) at 354 nm. Drug-free samples were used as controls, and measurements were conducted in triplicate.

### 2.8. Antimicrobial Study

Antimicrobial studies were conducted as described previously [52]. In brief, Gram-negative *Escherichia coli* (ATCC 25922) and Gram-positive *Staphylococcus aureus* (ATCC 29213) were spread evenly over the surface of LB agar plates, and dry or pre-hydrated sample punches (6 mm in diameter) or 30 µg T discs as a control were placed on top of the plates. After 24 h incubation at 37 °C, images of the inhibition zones were recorded with ChemiDoc imaging system (BioRad Laboratories, Copenhagen, Denmark) and analyzed with ImageJ software (1.52a version). All experiments were performed in triplicate (biological replicates).

### 2.9. In Vitro Cell Cultures

Primary neonatal human dermal fibroblasts (Invitrogen) were cultured following the manufacturer’s instructions. For the MTT and lactate dehydrogenase (LDH) assays, 5 × 10^4^ cells in 1 mL were seeded in each well of a 24-well plate and cultured to 90% confluence. Sample punches (6 mm diameter) were placed in the wells, while untreated cells and cells with 30 µL T solution were used as negative and positive controls, respectively, followed by incubation for 24 h at 37 °C and 5% CO_2_. Additionally, aqueous solutions of PDA (0.5 mg mL^−1^, 0.6 mg mL^−1^ and 0.9 mg mL^−1^) and PEI (0.6 mg mL^−1^, 1.1 mg mL^−1^ and 2.2 mg mL^−1^) were used as control samples.

### 2.10. Cell Viability Assays

LDH release in the cell culture medium was measured using the Pierce LDH Cytotoxicity Assay kit (Thermo Fisher, Roskilde, Denmark), according to the manufacturer’s instructions. For the MTT assay, the samples were removed and the remaining medium was replaced with 100 µL of fresh medium and 11 µL of MTT solution (5 mg mL^−1^ in PBS); lysed cells were used as a control. After 2–4 h incubation, cells were washed with PBS and 100 µL of dimethyl sulfoxide was added followed by a 10 min incubation at RT and measurement of the absorbance at 550 nm using a VICTOR Nivo plate reader (Perkin Elmer, Skovlunde, Denmark). All experiments were carried out in triplicate (biological replicates), except for the experiments with the PDA and PEI control solutions, which were only performed once for each solution.

### 2.11. Statistical Analysis

All data were analyzed in Origin software (version 9.6.0.172, OriginLab Corporation, Northampton, MA, USA). Tukey’s test was used for mean comparison after one-way analysis of variance and Levene’s test for equality of variances. The data are presented as means with standard deviations of *p* < 0.0332 (*), *p* < 0.0021 (**), *p* < 0.0002 (***) and *p* < 0.0001 (****). 

## 3. Results

### 3.1. Morphological Characterization of Nanofiber Scaffolds

**Figure 2:**
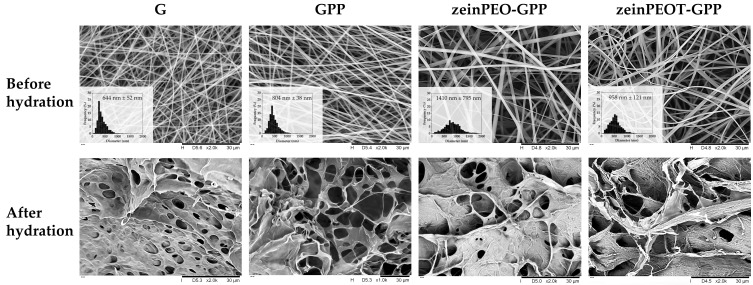
SEM analysis of the fiber morphology and diameter for different fiber scaffolds freshly prepared and after 24 h exposure to Milli-Q water and freeze-drying, respectively. Key: G, sample containing gelatin; GPP, sample containing gelatin crosslinked by polydopamine and polyethyleneimine; zeinPEO-GPP, sample containing zein and polyethylene oxide in the core and gelatin crosslinked by polydopamine and polyethyleneimine in the shell; zeinPEOT-GPP, sample containing zein, polyethylene oxide and tetracycline hydrochloride in the core and gelatin crosslinked by polydopamine and polyethyleneimine in the shell.

Electrospinning gelatin in AA yielded tubular fibers (sample G), and the addition of PEI and dopamine HCl to gelatin significantly increased the fiber diameter (*p* < 0.0001) (sample GPP) (Figure 2). The coaxially electrospun zein-containing fibers zeinPEO-GPP and zeinPEOT-GPP showed significantly larger fiber diameters (*p* < 0.0001) in comparison to all uniaxially electrospun samples. Interestingly, the incorporation of T led to a significant reduction (*p* < 0.0001) in fiber diameter compared to zeinPEO-GPP (Figure 2). 

### 3.2. Interaction of Nanofiber Scaffolds with Water

Uniaxially and coaxially electrospun scaffolds demonstrated different behavior upon hydration. While both types experienced morphological transformation from nanofibers into hydrogels with large pores, zeinPEO-GPP and zeinPEOT-GPP still contained visible nanofibers throughout the hydrogel structure that had formed during hydration (Figure 2). 

**Figure 3:**
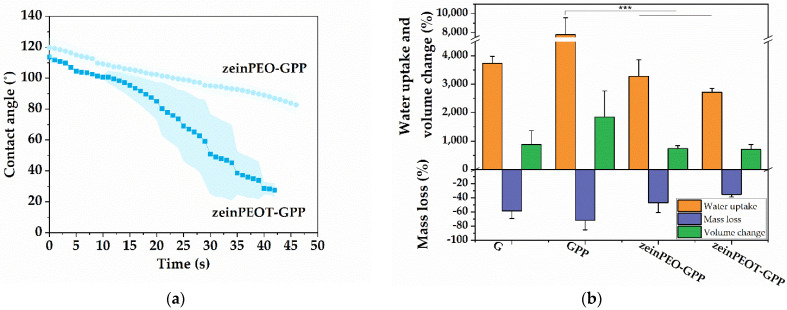
Interactions of nanofiber scaffolds with water. (**a**) Water contact angle (wettability) and (**b**) water uptake, volume change and mass loss after 24 h of incubation of the nanofiber scaffolds in Milli-Q water and their transformation into hydrogels. The data are presented as means with standard deviations of *p* < 0.0002 (***). Key: G, sample containing gelatin; GPP, sample containing gelatin crosslinked by polydopamine and polyethyleneimine; zeinPEO-GPP, sample containing zein and polyethylene oxide in the core and gelatin crosslinked by polydopamine and polyethyleneimine in the shell; zeinPEOT-GPP, sample containing zein, polyethylene oxide and tetracycline hydrochloride in the core and gelatin crosslinked by polydopamine and polyethyleneimine in the shell.

The wettability (water contact angle) experiments over 50 s on dry samples showed that the uniaxially electrospun samples G and GPP immediately absorbed the water droplet, making it impossible to measure contact angles. In comparison, contact angles for the coaxial samples zeinPEO-GPP and zeinPEOT-GPP were ~120° initially. While the water droplet was slowly absorbed into the zeinPEO-GPP scaffolds, it was more quickly absorbed in the case of their drug-loaded counterparts (zeinPEOT-GPP) (Figure 3a). 

**Figure A1:**
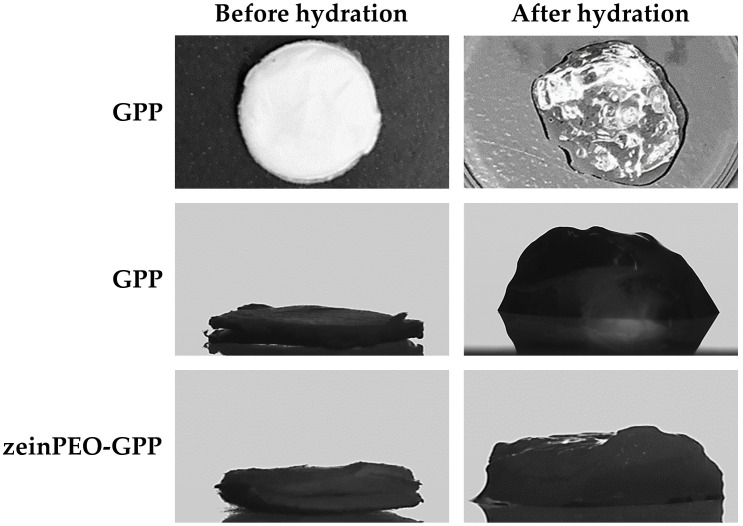
Volume change of 6 mm sample punches of GPP and zeinPEO-GPP upon hydration with water and transformation into a swollen hydrogel. Key: G, sample containing gelatin; GPP, sample containing gelatin crosslinked by polydopamine and polyethyleneimine; zeinPEO-GPP, sample containing zein and polyethylene oxide in the core and gelatin crosslinked by polydopamine and polyethyleneimine in the shell.

Incubation of the samples in water for 24 h led to substantial physical swelling of all tested samples (Figure A1), as confirmed by their water uptake between ~2500% (zeinPEOT-GPP) and 8000% (GPP) as well as volume change between ~800% (zeinPEO-GPP and zeinPEOT-GPP) and 2000% (GPP) (Figure 3b). Mass losses between 40% (zeinPEOT-GPP) and 80% (GPP) were found, with the highest mass loss being detected for GPP, which also showed the highest swelling (Figure 3b). Moreover, a water vapor sorption of approximately 45% and 33% was found for the uniaxially and coaxially electrospun scaffolds, respectively (Table 2).

The pore analysis revealed pore sizes of 3.0 µm ± 2.3 µm (G), 9.2 µm ± 4.9 µm (GPP), 36.2 µm ± 6.8 µm (zeinPEO-GPP) and 26.3 µm ± 4.7 µm (zeinPEOT-GPP) for the different samples after 24 h of hydration.

### 3.3. Mechanical Characterization

The results of the mechanical characterization are summarized in Table 2. Tensile tests were only conducted on the dry samples due to the large swelling of the samples in water. The coaxially electrospun scaffolds zeinPEO-GPP and zeinPEOT-GPP were slightly more elastic, i.e., showed lower Young’s moduli, and demonstrated lower strengths than the uniaxially electrospun samples G and GPP. The elongation at break was comparable for all tested samples and between 2.5% and 5.2%. 

Texture profile analysis was carried out to determine the compression behavior of the samples, including hardness, springiness and cohesiveness (Table 2). Analysis was only possible for the coaxially electrospun samples zeinPEO-GPP and zeinPEOT-GPP as the uniaxially spun samples G and GPP were too fragile. ZeinPEO-GPP and zeinPEOT-GPP showed good compressibility and ability to spring back to their original height after the first compression (springiness ~100%), while only ~60% was reached after the second compression (cohesiveness). The hardness was found to be similar for both the drug-loaded and the drug-free samples. 

### 3.4. Solid-State Characterization

**Figure 4:**
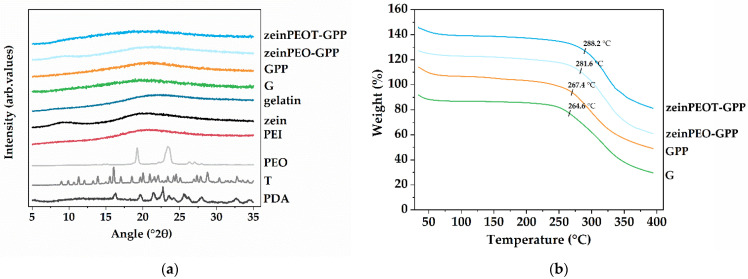
Solid-state characterization of the nanofiber scaffolds. (**a**) X-ray diffractograms of different nanofiber scaffolds and the raw materials used to prepare them and (**b**) TGA data of different nano-fiber scaffolds. Key: PDA, polydopamine; T, tetracycline hydrochloride; PEO, polyethylene oxide; PEI, polyethyleneimine; G, fiber sample containing gelatin; GPP, fiber sample containing gelatin crosslinked by polydopamine and polyethyleneimine; zeinPEO-GPP, fiber sample containing zein and polyethylene oxide in the core and gelatin crosslinked by polydopamine and polyethyleneimine in the shell; zeinPEOT-GPP, fiber sample containing zein, polyethylene oxide and tetracycline hydrochloride in the core and gelatin crosslinked by polydopamine and polyethyleneimine in the shell.

XRD patterns of all analyzed samples show a halo characteristic of amorphous materials, while some of the raw materials show characteristic peaks as they are crystalline (e.g., T) or semi-crystalline (e.g., PEO) (Figure 4a). The TGA results show two degradation steps, where the first, between 50 °C and 100 °C, can be attributed to water loss (Figure 4b). The onset of sample degradation was determined to be 273.0 °C ± 2.7 °C (G), 266.6 °C ± 0.9 °C (GPP), 282.9 °C ± 2.1 °C (zeinPEO-GPP) and 286.5 °C ± 1.5 °C (zeinPEOT-GPP), with the onset of degradation of the core-shell fiber being slightly higher than those of the uniaxial samples (*p* < 0.001) (Table A1).

### 3.5. Drug Loading and Release

**Figure 5:**
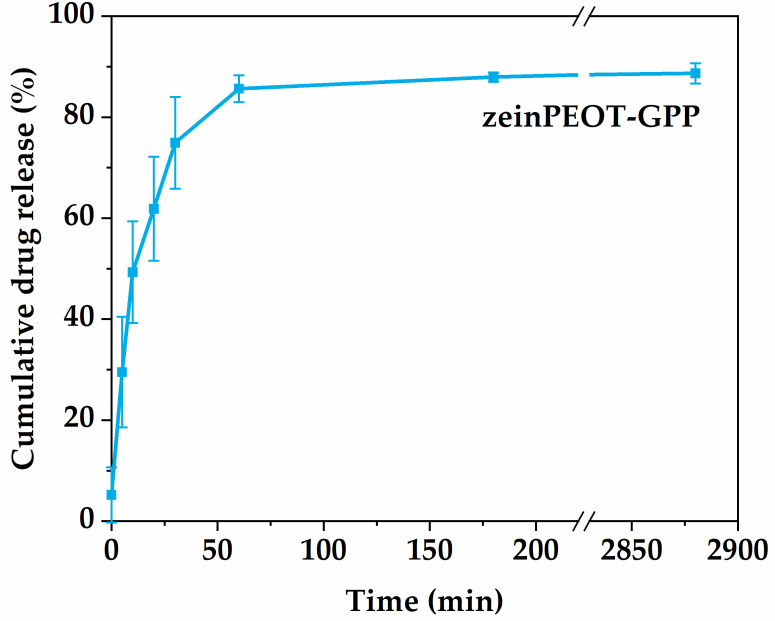
Cumulative release from zeinPEOT-GPP scaffolds. Key: zeinPEOT-GPP, fiber sample containing zein, polyethylene oxide and tetracycline hydrochloride in the core and gelatin crosslinked by polydopamine and polyethyleneimine in the shell.

The encapsulation efficiency of T within zeinPEOT-GPP scaffolds reached 72.2 ± 6.2%. A burst release of T was observed within an hour from zeinPEOT-GPP scaffolds (Figure 5). The release kinetics of T followed the Korsmeyer–Peppas model (Table A2) [53].

### 3.6. Biological Studies

**Figure 6:**
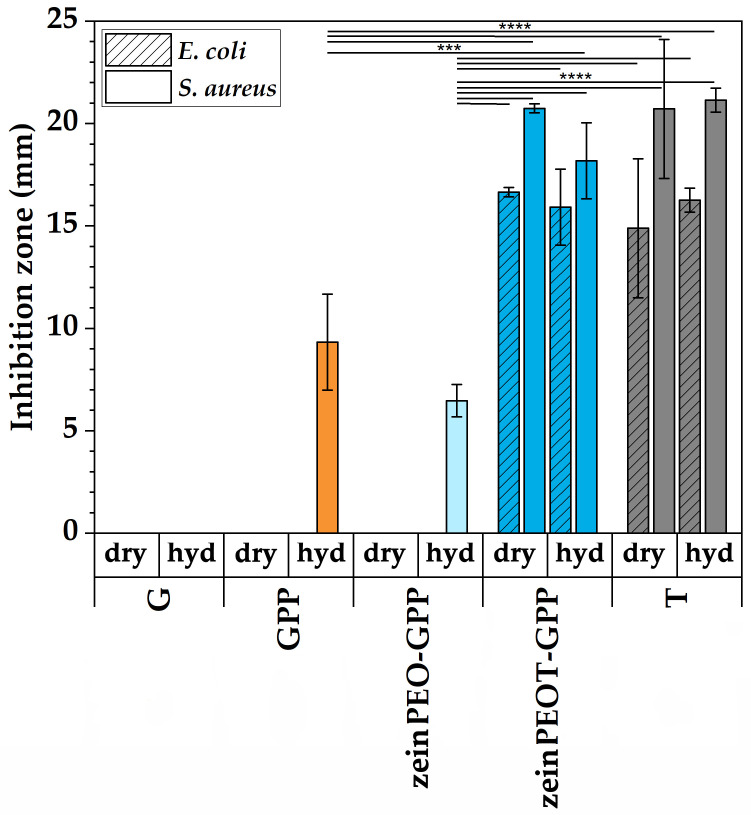
Results for the antimicrobial studies. Bacterial inhibition of *E. coli* and *S. aureus* by dry and pre-hydrated (hyd) nanofiber scaffolds. The data are presented as means with standard deviations of *p* < 0.0002 (***) and *p* < 0.0001 (****). Key: G, fiber sample containing gelatin; GPP, fiber sample containing gelatin crosslinked by polydopamine and polyethyleneimine; zeinPEO-GPP, fiber sample containing zein and polyethylene oxide in the core and gelatin crosslinked by polydopamine and polyethyleneimine in the shell; zeinPEOT-GPP, fiber sample containing zein, polyethylene oxide and tetracycline hydrochloride in the core and gelatin crosslinked by polydopamine and polyethyleneimine; T, tetracycline hydrochloride-containing control sample.

**Figure A2:**
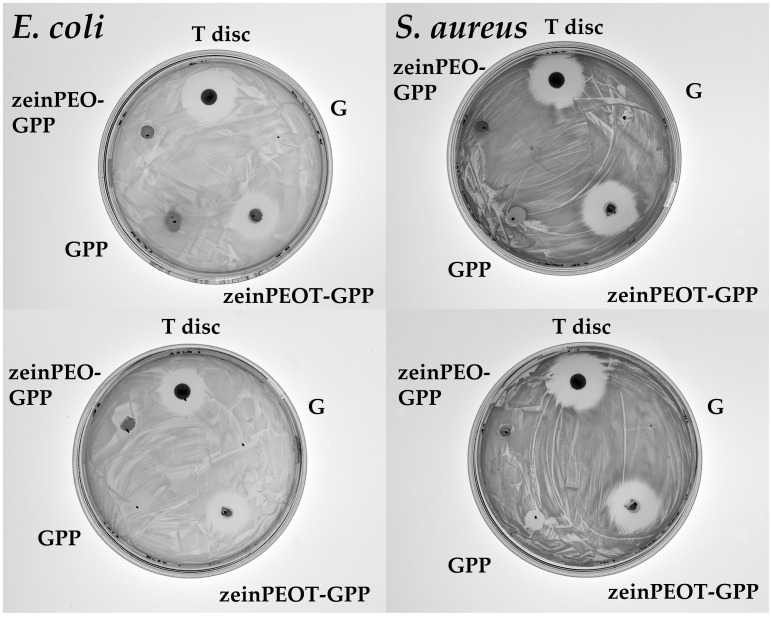
Results of the agar diffusion test of uniaxially and coaxially electrospun nanofiber scaffolds. Key: G, sample containing gelatin; GPP, sample containing gelatin crosslinked by polydopamine and polyethyleneimine; zeinPEO-GPP, sample containing zein and polyethylene oxide in the core and gelatin crosslinked by polydopamine and polyethyleneimine in the shell; zeinPEOT-GPP, sample containing zein, polyethylene oxide and tetracycline hydrochloride in the core and gelatin crosslinked by polydopamine and polyethyleneimine in the shell; T, tetracycline hydrochloride control sample.

Agar diffusion tests demonstrated contact-based inhibition of bacterial cultures of pre-hydrated T-free samples GPP and zeinPEO-GPP, i.e., samples containing PEI, while dry GPP and zeinPEO-GPP samples showed no inhibition of bacterial cultures (Figure 6). From the agar plates, it may be assumed that there was bacterial inhibition present initially, but bacterial growth started again at a later point (Figure A2). In contrast, clear inhibition zones were observed both for dry and pre-hydrated T-containing samples (Figure 6).

**Figure 7:**
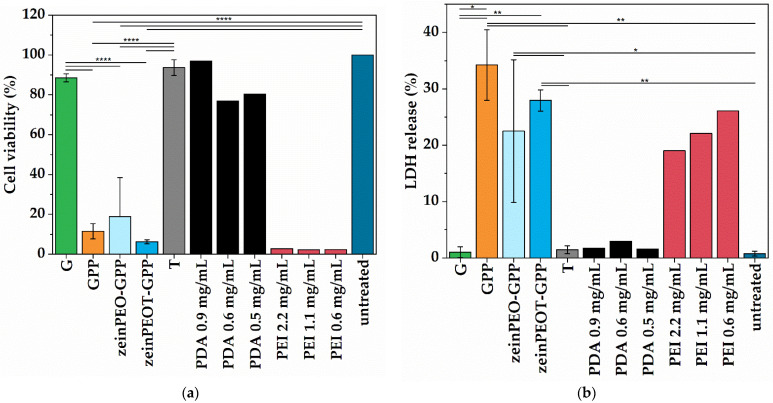
Results for the cell viability studies. (**a**) MTT assay and (**b**) LDH assay for human dermal fibroblasts in the presence of different nanofiber scaffolds or aqueous PDA and PEI solutions in different concentrations, respectively. The data are presented as means with standard deviations of *p* < 0.0332 (*), *p* < 0.0021 (**) and *p* < 0.0001 (****). Key: G, fiber sample containing gelatin; GPP, fiber sample containing gelatin crosslinked by polydopamine and polyethyleneimine; zeinPEO-GPP, fiber sample containing zein and polyethylene oxide in the core and gelatin crosslinked by polydopamine and polyethyleneimine in the shell; zeinPEOT-GPP, fiber sample containing zein, polyethylene oxide and tetracycline hydrochloride in the core and gelatin crosslinked by polydopamine and polyethyleneimine in the shell; T, tetracycline hydrochloride control sample; PDA, polydopamine; PEI, polyethyleneimine.

All samples containing PEI, including the PEI control samples in two concentrations, demonstrated high cytotoxicity towards fibroblasts according to both LDH and MTT assays (Figure 7). In contrast, sample G as well as the T, PDA and untreated control samples showed good cell viability above 80% (Figure 7a) and a low LDH release (Figure 7b).

## 4. Discussion

In our study, we achieved a successful transformation of all electrospun samples from two-dimensional nanofibrous scaffolds into three-dimensional hydrogels upon contact with water (Figure 2 and Figure A1). While the uniaxially electrospun fiber scaffolds (samples G and GPP) completely turned into hydrogels, hydrogels containing nanofibers (nanofiber-reinforced hydrogels) were formed from the coaxially electrospun core-shell scaffolds (samples zeinPEO-GPP and zeinPEOT-GPP) (Figure 2). The formation of nanofiber-reinforced hydrogels from the latter samples is associated with crosslinked gelatin swelling in water, while hydrophobic zein neither takes up much water nor dissolves in water, hence remaining intact, consistent with our previous work on zein fiber scaffolds and that of other studies [9,34]. In contrast, transformation of the uniaxially electrospun gelatin nanofibers (samples G and GPP) was found to be associated with a higher water uptake, volume change and mass loss as compared to the coaxially electrospun nanofibers (zeinPEO-GPP and zeinPEOT-GPP) (Figure 3b). Since no zein was present in the uniaxial samples, the large water uptake during complete transformation of these samples into hydrogels with no stabilizing fibers present makes the hydrogel mechanically weaker, i.e., prone to tear, as has been described previously for hydrogel systems [2,9,54]. As described earlier, for skin regeneration purposes, a high enough pore size is relevant for cell migration into the scaffold and skin regeneration. We found the highest pore sizes of ~30 µm for our nanofiber-reinforced hydrogels, which is beneficial for migration of fibroblasts (3–15 µm in diameter [55]).

As mentioned above, the mass loss of all scaffolds may be associated with incomplete crosslinking and potentially dissolution of gelatin and leaking of PDA and PEI from the fiber shell. In our study, the formation of PDA was induced by decomposition of (NH_4_)_2_CO_3_ to release NH_3_ vapors, which act as an oxidant on dopamine HCl [41] and induce rearrangement of dopamine into different quinone structures, such as 5,6-dihydroxyindole, as well as the formation of self-assembled trimers of (dopamine)_2_/5,6-dihydroxyindole [56,57]. With respect to interaction with PEI and gelatin, it has been described that after formation of PDA, PEI is covalently linked to the polymer [45] and that PDA and PEI crosslink with gelatin [38,41]. Overall, however, it is unclear how efficient these crosslinking processes are as they are most likely determined by many factors, including the penetration depth of the NH_3_ vapors. It seems possible, that crosslinking in our study predominantly occurred on the surface and in a non-exhaustive fashion, which is in accordance with a previous study, which confirmed the presence of free PEI in electrospun samples [41].

With respect to the contact angle (wettability), surface properties, including microstructure and hydrophilicity of the fiber scaffolds are of importance. While water was absorbed instantaneously by the uniaxially electrospun scaffolds (G, GP, GPP and TGPP), the coaxial scaffolds (zeinPEO-GPP and zeinPEOT-GPP) did not fully absorb water droplets within 50 s. However, the contact angle decreased steadily (Figure 3a), which is in agreement with a study on uniaxial PDA-PEI-crosslinked gelatin fibers [38]. This slight difference in the wettability behavior may be due to the different electrospinning conditions. During coaxial electrospinning, a more pronounced stretching of the shell solution in comparison to uniaxial nanofibers is likely to have occurred due to a twice larger distance from the nozzle tip to the collector (Table 1). A higher jet stretching leads to a more constrained environment for the polymer in the shell solution and a slightly different interplay with the solvent, potentially changing the surface properties of the fibers as compared to the uniaxially electrospun scaffolds. In this case the constrained environment may have resulted in more non-polar groups orienting themselves towards to fiber surface, leading to higher contact angles as has been described earlier [58].

A biomaterial should show a high elasticity as well as a high tensile strength to allow for easy handling as well as resistance against wear and tear [3]. Both the dry uniaxial (G, GPP) and core-shell scaffolds (zeinPEO-GPP, zeinPEOT-GPP) demonstrated comparable low Young’s moduli (high elasticities) and high tensile strengths, which is beneficial for their use as biomaterials (Table 2). In fact, elasticities and tensile strengths are similar to those obtained for zein-polycaprolactone core-shell nanofibers developed by our group in an earlier study [52]. Importantly, these correspond well with those of forearm skin [59], indicating that the materials may potentially be suited for tissue regeneration purposes after further optimization. However, analysis of the compression behavior was only possible for the core-shell fiber mats (zeinPEO-GPP and zeinPEOT-GPP), as the uniaxial samples (G, GPP) showed a too high fragility and loss of integrity during compression. Indeed, texture profile analysis of the samples confirmed good compressibility and hardness of zeinPEO-GPP and zeinPEOT-GPP, and the results obtained are comparable with previous findings for carboxymethyl chitosan–silk fibroin nanofiber-reinforced hydrogels [27].

Knowing the solid-state properties of nanofiber scaffolds in their dry state is important to estimate and understand their physical stability upon storage. The TGA data (Figure 4b) revealed three degradation steps, where the first corresponds to loss of absorbed and bound water [60], the second is related polymer degradation [61], and the third step corresponds to the final thermal decomposition of the sample. High stability of all samples > 90% of the original weight at the onset of sample degradation of ~270 °C was confirmed by TGA, with the zein-containing samples showing higher onsets of degradation in accordance with previous studies [62,63], while the onset of degradation for the uniaxial samples G and GPP correspond to reported degradation temperatures of gelatin powder [61]. Gelatin is an amorphous polymer, and the broad XRD patterns (Figure 4a) of the uniaxially electrospun samples (G, GPP) are in line with results obtained for gelatin reported earlier [60,64]. 

Antimicrobial biomaterials should release the loaded antibiotic in a controlled manner and thus inhibit bacterial growth. Approximately 76% of T was released from the zeinPEOT-GPP scaffolds within an hour (Figure 5). The release kinetics of T best followed the Korsmeyer–Peppas model, which describes drug release from hydrogel systems as a combined result of diffusion, swelling and subsequent erosion of the hydrogel [5,64]. With respect to an inhibition of bacterial growth, it was found that both zeinPEOT-GPP in dried and wetted states as well as T control samples effectively inhibited both investigated bacterial strains (Figure 6, Figure A2). The lower but pronounced antimicrobial effect of the pre-hydrated T-free scaffolds (GPP and zeinPEO-GPP) towards *S. aureus* is related to the presence of PEI, which has previously been reported to be effective, mostly against Gram-positive bacterial strains, such as *S. aureus* [41,44]. The fact that the dry scaffolds did not show any antimicrobial activity confirms a contact-based inhibition by PEI for the GPP and zeinPEO-GPP scaffolds. The loss of integrity of the GPP scaffold and, to a lesser extent, of zeinPEO-GPP upon hydration increased the antimicrobial effect as compared to the dry scaffolds because the sample spread further on the agar plate. Only a faint inhibition of *E. coli* was observed for pre-hydrated GPP and zeinPEO-GPP (Figure A2), most likely due to the insufficient inhibition of *E. coli* by PEI. It has indeed been shown in other studies that higher concentrations of PEI are necessary to inhibit *E. coli* compared to *S. aureus* [41,44].

Fibrous as well as hydrogel biomaterials should demonstrate biocompatibility [4,13,30]. The high cytotoxicity observed in the LDH and MTT assays for all samples, except for the samples from pristine G (Figure 7a,b), revealed that the nanofiber scaffolds require further optimization before they can be used for tissue regeneration. The cytotoxicity of the scaffolds is due to the presence of PEI as confirmed by pure PEI control samples. Various approaches are possible for an improving the cytocompatibility of the nanofiber scaffolds including using linear PEI or optimizing the crosslinking of branched PEI by increasing the exposure time to (NH_4_)_2_CO_3_ [41]. Moreover, PEI can be replaced by other cationic polymers [65] providing a high antimicrobial effect and acceptable cell toxicity, such as antimicrobial polypeptides (ε-polylysine [66]) or biodegradable antimicrobial polymers (cationic polycarbonates [67]). For instance, it has been shown in a previous study that a hybrid nanofibrous matrix composed of polycaprolactone and poly(citrate)-ε-polylysine displays biomimetic elastomeric properties, robust antibacterial activity and excellent biocompatibility [66]. Another option is to replace PEI with other biocompatible crosslinkers without antimicrobial effect that have been described for gelatin, including genipin or transglutaminase [68]. While further work is needed to allow our fiber-reinforced hydrogels to be used in skin regeneration, the present study nevertheless provides a proof-of-concept demonstration that such materials may indeed be conveniently manufactured in a one-step approach allowing scale-up in production.

## 5. Conclusions

This study provides proof of concept for the in situ transformation of core-shell nanofibers produced by coaxial electrospinning into nanofiber-reinforced hydrogels. The core-shell nanofibers provide favorable mechanical properties, efficient water uptake and pronounced swelling as well as good compressibility. Taken together, these properties represent first steps towards the use of the nanofiber scaffolds in skin regeneration. However, while potent antimicrobial activity of the nanofiber-reinforced hydrogels was observed both due to the immediate release of T and the activity of PEI, cytotoxicity towards fibroblasts was found to be induced by PEI and represents a problem. Hence, further work is needed to reduce toxicity of the nanofiber scaffolds and the in situ-forming nanofiber-reinforced hydrogels before they can be tested and used for skin regeneration purposes. 

## Figures and Tables

**Table 1:** Sample description and electrospinning settings.

Sample	Core	Shell	Flow Rate Core, µL h^−1^	Flow Rate Shell, µL h^−1^	Injector Voltage, kV	Collector Voltage, kV	Distance, cm
G	Gelatin	-	250	-	8	−1	12
GPP	Gelatin, PEI, PDA	-	250	-	9	0	12
zeinPEO-GPP	Zein, PEO	Gelatin, PEI, PDA	400	400	9	−7.5	21.5
zeinPEOT-GPP	Zein, PEO, T	Gelatin, PEI, PDA	400	400	9	−6.5	21.5

**Table 2:** Tensile properties, compression behavior and water vapor sorption capacity of the dry nanofiber scaffolds.

Sample	Young’s Modulus, kPa	Tensile Strength, kPa	Elongation at Break, %	Vapor Sorption, %	Hardness, N	Cohesiveness, %	Springiness, %
G	18.9 ± 13.5	490.3 ± 286.8	4.2 ± 0.2	48.4	-	-	-
GPP	31.6 ± 1.2	527.7 ± 182.2	2.6 ± 0.4	45.4	-	-	-
zeinPEO-GPP	4.8 ± 4.9	131.1 ± 126.6	5.2 ± 2.2	33.3	0.65 ± 0.17	65.0 ± 8.6	99.9 ± 1.3
zeinPEOT-GPP	4.9 ± 3.0	97.5± 63.9	3.2 ± 0.4	33.9	0.46 ± 0.17	55.6 ± 4.3	100.3 ± 1.3

## Data Availability

Not applicable.

## Abbreviations

AA, acetic acid; LB, Luria–Bertani; LDH, lactate dehydrogenase; MTT, 3-(4,5-dimethylthiazol-2-yl)-2,5-diphenyltetrazolium bromide; OD, optical density; PBS, phosphate-buffered saline; PDA, polydopamine; PEI, polyethyleneimine; PEO, polyethylene oxide; RGD, arginine–glycine–aspartate; RT, room temperature; SEM, scanning electron microscopy; SD, standard deviation; T, tetracycline hydrochloride; TGA, thermogravimetric analysis; XRD, X-ray diffraction.

## Appendix A

**Table A1:** Onset of degradation temperatures for different samples determined from the TGA data. The data are presented as means with standard deviations of *p* < 0.0001 (****).

Sample	Onset of Degradation (°C)
G	273.0 ± 2.7
GPP	266.6 ± 0.9
zeinPEO-GPP	282.9 ± 2.1 **** (vs. G and GPP)
zeinPEOT-GPP	286.5 ± 1.5 **** (vs. G and GPP)

**Table A2:** Release kinetics of T from sample zeinPEOT-GPP.

Sample	Model Fitting			
	Zero Order R^2^	First Order R^2^	Higuchi R^2^	Korsmeyer–Peppas R^2^
zeinPEOT-GPP	0.901	0.708	0.983	0.991 (*n* = 0.48)
